# Functional and RNA-Sequencing Analysis Revealed Expression of a Novel Stay-Green Gene from *Zoysia japonica* (*ZjSGR*) Caused Chlorophyll Degradation and Accelerated Senescence in Arabidopsis

**DOI:** 10.3389/fpls.2016.01894

**Published:** 2016-12-16

**Authors:** Ke Teng, Zhihui Chang, Xiao Li, Xinbo Sun, Xiaohong Liang, Lixin Xu, Yuehui Chao, Liebao Han

**Affiliations:** ^1^Turfgrass Research Institute, Beijing Forestry UniversityBeijing, China; ^2^Institute of Animal Science, Chinese Academy of Agricultural SciencesBeijing, China; ^3^Key Laboratory of Crop Growth Regulation of Hebei Province, Agricultural University of HebeiBaoding, China

**Keywords:** *Zoysia japonica*, *SGR*s, senescence, chlorophyll degradation, RNA sequencing

## Abstract

Senescence is not only an important developmental process, but also a responsive regulation to abiotic and biotic stress for plants. Stay-green protein plays crucial roles in plant senescence and chlorophyll degradation. However, the underlying mechanisms were not well-studied, particularly in non-model plants. In this study, a novel stay-green gene, *ZjSGR*, was isolated from *Zoysia japonica*. Subcellular localization result demonstrated that ZjSGR was localized in the chloroplasts. Quantitative real-time PCR results together with promoter activity determination using transgenic Arabidopsis confirmed that *ZjSGR* could be induced by darkness, ABA and MeJA. Its expression levels could also be up-regulated by natural senescence, but suppressed by SA treatments. Overexpression of *ZjSGR* in Arabidopsis resulted in a rapid yellowing phenotype; complementary experiments proved that *ZjSGR* was a functional homolog of *AtNYE1* from *Arabidopsis thaliana*. Over expression of *ZjSGR* accelerated chlorophyll degradation and impaired photosynthesis in Arabidopsis. Transmission electron microscopy observation revealed that overexpression of *ZjSGR* decomposed the chloroplasts structure. RNA sequencing analysis showed that *ZjSGR* could play multiple roles in senescence and chlorophyll degradation by regulating hormone signal transduction and the expression of a large number of senescence and environmental stress related genes. Our study provides a better understanding of the roles of *SGR*s, and new insight into the senescence and chlorophyll degradation mechanisms in plants.

## Introduction

Senescence is not only an important developmental process, but also a responsive regulation to abiotic and biotic stress for plants (Wingler and Roitsch, 2008). Senescence of turfgrass and forage is of particular interest because the market value of these widely planted species are generally determined by the visual quality, nutritional value, and biomass accumulation. The most obvious phenomenon in senescence development is the change of leaf colors that usually turn from green to yellowish or red (Zhou et al., 2011). The underlying mechanism of senescence is far more comprehensive than the simple leaf color changes. It has been reported that senescence is highly programmed, including coordinated changes in cell structure, metabolism and genetic manipulation (Hörtensteiner, 2006; Schippers et al., 2015). By ultrastructual analysis, chloroplasts were proved to be the first organelles to be dismantled in senescence process (Dodge, 1970). Because the chloroplasts contain the majority of leaf protein that are attuned to be recycled during senescence in plants, the metabolic changes in chloroplasts may lead to drastic downstream regulation, suggesting the essential role of chloroplast disassembly in senescence (Jibran et al., 2015; Lin et al., 2015). Chlorophyll degrades via the multistep pheophorbide *a* oxygenase (PAO) pathway during senescence, comprising several chloroplast-located reactions (Christ and Hörtensteiner, 2014).

The STAY-GREEN (SGR) proteins function independently of PAO as key regulators in chlorophyll degradation (Hörtensteiner, 2006). Among the five identified *SGR*s mutants (A–E), the type C mutant with delayed chlorophyll degradation but normal decline rate of photosynthesis are the best characterized and utilized to further study the mechanism of chlorophyll degradation and senescence (Jibran et al., 2015). It has been identified from *Lolium perenne* (MacDuff et al., 2002), *Oryza sativa* (Park et al., 2007), *Arabidopsis thaliana* (Ren et al., 2007) and *Medicago truncatula* (Zhou et al., 2011). Sequence comparison results prove that SGRs are highly conserved proteins without any characterized domain, leading to the hypothesis that *SGR*s encode regulatory proteins regulating chlorophyll degradation rather than function as one particular enzyme (Hörtensteiner, 2006; Park et al., 2007). Functional studies suggest that SGRs are likely to function in dismantling of chlorophyll-apoprotein complexes and its expression could be a prerequisite for chlorophyll degradation in senescence (Hörtensteiner, 2006). Moreover, previous studies show that SGRs could play multiple roles in senescence besides chlorophyll degradation (Zhou et al., 2011; Christ and Hörtensteiner, 2014). Nevertheless, due to the limitation of traditional molecular biology approaches, the global picture of the regulatory networks of *SGR*s are not yet well-elucidated (Park et al., 2007; Zhou et al., 2011). What's more, the study of chlorophyll degradation and senescence in the non-model plants lags far behind that in model plants.

To date, many emerging molecular techniques have been applied to explore the underlying mechanisms of plant senescence. Microarray analysis of Arabidopsis leaf developmental processes, *M. truncatula stay-green* mutants, and *Populus tremula* revealed that senescence in plant was a rather complex system (Andersson et al., 2004; Breeze and Buchanan-Wollaston, 2011; Zhou et al., 2011). RNA-seq was utilized in *Gossypium hirsutum* to better understand the leaf development and senescence, and it provided the first most comprehensive dataset for cotton leaf senescence (Lin et al., 2015). Chao et al. (unpublished) took advantage of the single-molecule real-time long-read isoform sequencing to explore the genetic regulatory network of leaf senescence in *Medicago sativa*. However, the global gene expression analysis focused on plant senescence is currently limited compared with that focused on biotic and abiotic stresses. Moreover, the underlying transcriptome dynamics are not well-elucidated due to the limited transcript profiling data of different species (Andersson et al., 2004; Breeze and Buchanan-Wollaston, 2011; Zhou et al., 2011). Therefore, it is vital to enrich the transcript profiles to better understand the senescence process at the transcriptional level and to further explore the functional roles of *SGRs* in senescence regulatory networks.

*Zoysia japonica* is a widely used warm-season C_4_ turfgrass species which owns many excellent characters, such as low maintenance requirements, good traffic tolerance and excellent tolerance to heat, drought, and salinity stresses (Patton and Reicher, 2007; Xu et al., 2015; Teng et al., 2016). However, the shorter green period of *Z. japonica* compared with cool season turfgrass is becoming a prominent barrier preventing its market promotion in the transition zone and the northern regions. It has been a desire of the turfgrass breeders for a long time to cultivate zoysiagrass cultivars with longer green period and better visual appearance. Nevertheless, limited genetic resources are available for zoysiagrass to date (Wei et al., 2015; Tanaka et al., 2016), and the senescence mechanism are far from being well-illustrated. The objectives of this study were: (i) to identify a stay-green protein gene in *Z. japonica*; (ii) to investigate its characters and explore its functional roles; and (iii) to further explore its regulatory network in senescence. Thus, *ZjSGR* was isolated and characterized from *Z. japonica*. The gene expression character, subcellular localization pattern, and functional roles were studied. Furthermore, the underlying regulatory mechanisms of *ZjSGR* were investigated using RNA sequencing (RNA-seq) in *ZjSGR*-overexpressing Arabidopsis. This study will provide new insight into plant senescence induced by SGRs.

## Materials and methods

### Plant materials and growth conditions

*Z. japonica* cultivar “Zenith” were planted in a greenhouse with temperature at 28/25°C (day/night) with a 14 h light period. *A. thaliana* ecotype Col-0 and *nye1-1* (*stay-green*) mutant were used to generate transgenic lines. *A. thaliana* plants were cultivated in the nutrition medium containing peat, vermiculite and pearlite (1:1:1 in volume) and were kept at 24/22°C (day/night) with 16 h photoperiod and 65% humidity in growth chambers. *Nicotiana benthamiana* were cultivated in a growth chamber maintained at 24°C with a 16 h photoperiod. Half-strength Hoagland's solution was used weekly to fertilize the plants (Hoagland and Arnon, 1950).

### Isolation of *ZjSGR* and its promoter

Total RNA and genomic DNA were isolated from “Zenith” leaves of 3-month-old using the Trizol method (Invitrogen, USA), and the CTAB method, respectively. Using PrimeScript RT Master Mix (TaKaRa, Japan), the cDNA was obtained with total RNA as template. Using the ZjSGR-F and ZjSGR-R (Table 1), the cDNA sequence and gDNA sequences of *ZjSGR* were amplified. To obtain the promoter sequence of *ZjSGR*, TAIL PCR was carried out using a Genome Walking Kit (TaKaRa, Japan) with genomic DNA as template. Three gene specific primers, SGR-R1, SGR-R2, and SGR-R3 (Table 1), were used in the chromosome walking. Based on the sequencing data, the promoter-specific primers, Promoter-F, and Promoter-R, were synthesized to amplify the upstream sequence of *ZjSGR* (Table 1).

**Table 1:** **Primers used for gene cloning, qRT-PCR detection and plasmid construction**.

**Primer name**	**Primer sequence (5′-3′)**
ZjSGR-F	CCAGGAGAAGGGAAGGCGCGAACAT
ZjSGR-R	TTGTCATCACCGGTCCCCGTGTCAC
SGR-R1	CTTCCGGAAGATGTAGTACCGGAGG
SGR-R2	GCAGCGACATCCGGCCGCGCACCTT
SGR-R3	CGGTTGTACCACCCCTGCAGCTGCGCG
Promoter-F	CAACCACTGTGACTTGGAAGTTATG
Promoter-R	CTGCTTGAGCTGGGAGATCTG
ZjACT-F	GGTCCTCTTCCAGCCATCCTTC
ZjACT-R	GTGCAAGGGCAGTGATCTCCTTG
qSGR-F	CGTCCACTGCCACATCTCCG
qSGR-R	CGAACGCCTTCAGCACCACA
3302Y-SGR-F	cacgggggactcttgaccatggtaATGGCTGCGGCCATTTCGG
3302Y-SGR-R	ggtacacgcgtactagtcagatcCTGCTGCGTCTGGCCAGCG
3302FLAG-SGR-F	gagaacacgggggactcttgacATGGCTGCGGCCATTTCGG
3302FLAG-SGR-R	ccttgtaatccagatctaccatCTGCTGCGTCTGGCCAGCG
3302GUS-SGR-F	aagcctagggaggagtccacCAACCACTGTGACTTGGAAG
3302GUS-SGR-R	tttaccctcagatctaccatGTTCGCGCCTTCCCTTCTC
Complement-F	aagcctagggaggagtccacCAACCACTGTGACTTGGAA
Complement-R	tttaccctcagatctaccatTTGTCATCACCGGTCCCCGT
AtrbcL-F	GGGTTCAAAGCTGGTGTTAAAG
AtrbcL-R	CTCGGAATGCTGCCAAGATA
AtPSAF-F	ACGGGAAGTACGGATTGTTATG
AtPSAF-R	CGATCCATCCAGCAATGTAGAG
AtCAB1-F	AGGAACCGTGAACTAGAAGTTATC
AtCAB1-R	CCGAACTTGACTCCGTTTCT
AtRCA-F	GTCCAACTTGCCGAGACCTAC
AtRCA-R	TTTACTTGCTGGGCTCCTTTT

### Quantitative real-time PCR analysis

Quantitative real-time PCR (qRT-PCR) analysis of leaves at different senescent stages and other tissues (roots and stems) were used to explore the expression pattern of *ZjSGR* in zoysiagrass. *ZjSGR* expression profiles were examined in 3-month-old zoysiagrass after 24 h induction with darkness, 10 μM ABA, 10 μM MeJA, or 0.5 mM SA. Reactions were performed in a 96-well blocks qRT-PCR system (CFX Connect, BIO-RAD, USA) using SYBR Premix (TaKaRa, Japan) in total volumes of 25 μL. The two-step qRT-PCR profile was set as follows: initial denaturing step of 95°C for 30 s, followed by 40 cycles of 95°C for 0.05 s, and 60°C for 30 s. The *Z. japonica beta-actin* was selected as the internal reference gene (GenBank accession No. GU290546) (Table 1). Gene specific primers, qSGR-F and qSGR-R (Table 1), used for qRT-PCR were designed based on the cDNA sequence. For the relative gene expression analysis in transgenic Arabidopsis, the *AtUBQ10* was selected as the internal reference, and all the related gene specific primers used in this study could be found in Table 1. The primers used for the RNA-seq data verification were listed in Table S1. The relative expression level was figured out using the comparative ΔΔ Ct method (Livak and Schmittgen, 2001). All data are presented as the means (with corresponding standard deviations, *SD*) of four independent RNA templates, each of which included four technical replicates.

### Construction of vectors and generation of transgenic Arabidopsis

The 35S::*ZjSGR:YFP* fusion construct was created by inserting the complete ORF of *ZjSGR* into plasmid 3302Y (Jia et al., 2016). Primers 3302Y-SGR-F and 3302Y-SGR-R (Table 1) were used to amplify the *ZjSGR* coding sequence (Table 1), which was then purified and inserted into 3302Y vector digested with *Bgl*II (TaKaRa, Japan) using an In-fusion HD Cloning Kit (TaKaRa, Japan). The 35S::ZjSGR:FLAG fusion construct was created by inserting the complete ORF of *ZjSGR* into plasmid 3302FLAG. Primers 3302FLAG-SGR-F and 3302FLAG-SGR-R (Table 1) were used to amplify the *ZjSGR* coding sequence (Table 1), which was then purified and inserted into 3302FLAG vector digested with *Nco*I (TaKaRa, Japan) using an In-fusion HD Cloning Kit (TaKaRa, Japan).

The *ZjSGR*_*pro*_*::GUS* fusion construct contained an 1029 bp *ZjSGR* 5′ upstream sequence. The promoter region was amplified from plasmid containing the target sequence using primers 3302GUS-SGR-F and 3302GUS-SGR-R (Table 1). The 3302GUS vector (without 35S promoter) was digested with *Nco*I (TaKaRa, Japan), and then the purified *ZjSGR* promoter was inserted into the digested vector using an In-fusion HD Cloning Kit (TaKaRa, Japan) to produce the *ZjSGR*_*pro*_*::GUS* fusion construct. To obtain the *ZjSGR*_*pro*_*::ZjSGR:GUS* construct, the target fragment was amplified using Complement-F and Complement-R, and then it was subcloned into the 3303GUS vector.

Using the floral dip method, *Agrobacterium* GV3101 containing the constructed plasmids were used to transform Arabidopsis plants to generate transgenic plants expressing *35S::ZjSGR:YFP* (for subcellular localization observation), *35S::ZjSGR:FLAG* (for overexpression assay), or *ZjSGR*_*pro*_*::GUS* (for promoter activity analysis) and *ZjSGR*_*pro*_*::ZjSGR:GUS* (for complementation test), respectively. Transformed Arabidopsis seeds were screened using 60 mg·L^−1^ glufosinate. Positive transgenic plants were verified by RT-PCR and genomic PCR. Representative T_3_ generation transgenic lines that exhibited 100% resistance to glufosinate were harvested for further phenotype observation and measurements.

### Phenotype observation and sampling time

The seedlings were cultivated in growth chambers for 4 weeks and then the phenotype was photographed using a digital camera (EOS 60D, Cannon, Japan) and the chlorophyll content, gene expression, and photosynthesis were determined. For the complementation test, the detached rosette leaves of independent complementary T_3_ lines were incubated in 3 mM MES buffer (pH 5.7) for 4 days in total darkness condition and then were photographed and sampled for gene expression level assay. For the promoter activity determination, T_3_ seedlings were cultivated in MS medium in the growth chamber for 2 weeks and then were kept in darkness or transplanted to the MS medium containing 10 μM ABA and 10 μM MeJA for 3 days, respectively. After that, the GUS assay was carried out according to the protocol of (Cervera, 2005).

### Subcellular localization of ZjSGR and GUS assay of *ZjSGR_*pro*_::GUS* transgenic seedlings

Using the stable transformed *ZjSGR*-overexpressing Arabidopsis, the subcellular localization character of ZjSGR was investigated using a laser confocal scanning microscope (SP-5, Leica, Germany). Using the stable transformed *ZjSGR*_*pro*_*::GUS* transgenic Arabidopsis lines, the GUS staining was carried out. After 3-day inducement under darkness and hormones, the seedlings were then photographed using a stereomicroscope (M205 FA, Leica, Germany).

### Determination of chlorophyll contents and photosynthesis

The chlorophyll content was measured according to the protocol of Knudson et al. (1977). In brief, leaf tissue (0.05 g) was immersed in 8 mL 95% ethanol in dark for 48 h, and the extraction was determined. The contents of chlorophyll a (Chl a) and chlorophyll b (Chl b) were measured with a spectrophotometer (UV-2802S, UNICO, Spain) by recording the absorbance at 649 and 665 nm. The chlorophyll content was calculated using the formulas: Chl (mg g^−1^ FW) = (6.63A_665_ + 18.08A_649_) × V/W, [A, optical density at 665 and 649 nm; V, final volume (milliliters); FW, leaf tissue fresh weight (grams)]. The net photosynthetic rate (Photo), stomatal conductance (Cond), intercellular-space CO_2_ concentration (Ci), and transpiration rate (Tr) were determined using photosynthesis system (Li6400XT, Li-Cor, USA) with a constant airflow rate of 500 μmol s^−1^ and ~400 μmol mol^−1^ CO_2_ concentration at 28°C.

### Chloroplast structure observation by transmission electron microscopy

Transmission electron microscopy (TEM) observation was carried out according to the method of Park et al. (2007) with minor modifications. Briefly, leaf tissues segments were fixed with a fixative buffer containing 2% glutaraldehyde, 2% paraformaldehyde, and 50 mM sodium cacodylate (pH 7.2). Then the fixed tissues were washed three times with 50 mM sodium cacodylate buffer (pH 7.2) at 4°C. The samples were incubated in 50 mM sodium cacodylate buffer containing 1% osmium tetroxide (pH 7.2) at 4°C for 2 h. Then the samples were washed twice with distilled water at 25°C, and immersed in 0.5% uranyl acetate for at least 30 min at 4°C, and then dehydrated in a gradient series of propylene oxide and ethanol before finally embedded in resin. Ultrathin sections were generated with an ultra-microtome and placed on copper grids after being polymerized at 70°C for 24 h. The samples on the grids were treated with 2% uranyl acetate for 5 min and with Reynolds′ lead citrate for 2 min at 25°C, and then photographed using a transmission electron microscope (HT7700, Hitachi, Japan).

### RNA-Seq analysis

Total RNA was extract from 3-week-old Arabidopsis using Trizol methods (Invitrogen, USA). RNA purity, concentration and integrity were determined with NanoPhotometer (IMPLEN, Germany), Qubit 2.0 Flurometer (Life Technologies, USA) and Bioanalyzer 2100 system (Agilent Technologies, USA), respectively. Three micrograms of RNA per sample was used to prepare RNA samples. Sequencing libraries were generated (3 μg RNA per sample) according to the manufacture's recommendations of NEBNext Ultra RNA Library Prep Kit for Illumina (NEB, USA). Index codes were added to attribute sequences to each sample. The clustering of the index-coded samples was performed on a cBot Cluster Generation System using TruSeq PE Cluster Kit v3-cBot-HS (Illumia, USA) according to the manufacturer's instructions. Then the libraries were sequenced on an Illumia HiSeq™ 4000 platform by Novogene Technologies (Beijing, China). Clean reads from the each library were mapped to reference Arabidopsis genome using TopHat v2.0.12 (Kim et al., 2013). The reads numbers mapped to each gene were counted using HTSeq v0.6.1 (Anders, 2015). FPKM was used to determine the gene expression levels. Genes with an adjusted *P* < 0.05 and absolute value of log_2_ FC ≥ 1 were assigned as differential expressed genes (DEGs) in the six libraries from *ZjSGR* transgenic plants (line SGR-7) and WT (3 libraries per group). For gene ontology (GO) enrichment analysis, all the DEGs were mapped to GO terms in the GO database (http://geneontology.org/). KOBAS software (Mao et al., 2005) was utilized to test the statistical enrichment of the DEGs in KEGG (http://www.kegg.jp/) pathways. The reliability of the RNA-seq data was confirmed by Pearson's correlation plot. The raw sequence reads were deposited into the National Center for Biotechnology Information (NCBI) Short Read Archive (SRA) repository under the accession number SRP093808.

### Western blot analysis

Total proteins were prepared from plant tissues using Trizol methods (Invitrogen, USA), and measured using the Bradford protein assay (BioRad, USA). Total protein (5 μg) samples were separated on a 12% SDS-PAGE, transferred to a pure nitrocellulose blotting membrane (PALL, USA), and consecutively probed with a Mouse mAb antibody (Cell Signaling Technology, USA) and an Anti-mouse IgG (Cell Signaling Technology, USA). The DAB Kit (SIGMA, Germany) was used to detect the immune complex.

### Statistical analyses

The data were analyzed statistically with Microsoft Excel 2010 (Microsoft, USA) and SPSS version 19 statistical software (SPSS Inc., USA).

## Results

### Isolation and characterization of the *SGR* gene and its promoter

The *ZjSGR* cDNA and gDNA sequences were deposited in the NCBI database with accession numbers KP148819 and KP148820, respectively. The ZjSGR protein consisted of 276 amino acids and belonged to the stay-green superfamily. A 1029 bp fragment of the ATG initial codon was obtained for promoter elements deduction and promoter activity assay. Multiple light responsive elements were identified, including AT1-motif, Box 1, Box 4, CATT-motif, MNF1, SP1, and TCT-motif (Table S2). Of particular interest, several elements involved in responses to phytohormone inducements were also detected, including CGTCA-motif (MeJA-responsiveness), TGACG-motif (MeJA-responsiveness), TCA-element (salicylic acid responsiveness), and TGA-element (auxin-responsive element; Table S2). These *cis*-regulatory elements encouraged us to explore the expression character of *ZjSGR* under phytohormone inducements.

**Figure 1:**
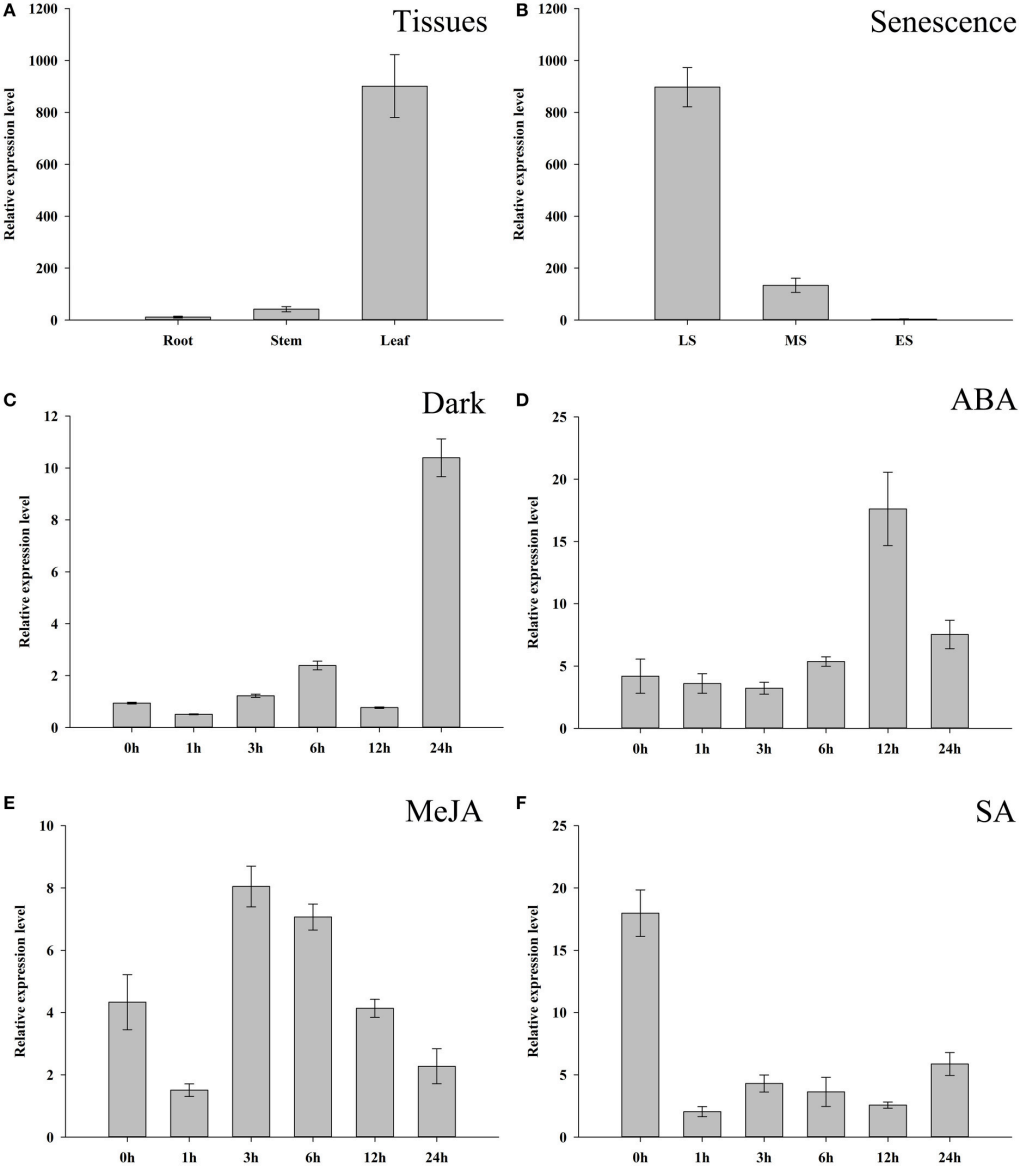
**Expression characters of ***ZjSGR***. (A)** *ZjSGR* expression levels in root, stem, and leaf of *Zoysia japonica*. **(B)** Expression levels of *ZjSGR* in *Z. japonica* leaves at different senescence levels. LS, late senescence; MS, middle senescence; ES, early senescence **(C–F)** *ZjSGR* expression levels in *Z. japonica* exposed to darkness **(C)**, 10 μM ABA **(D)**, 10 μM MeJA **(E)**, and 0.5 mM SA **(F)**.

**Figure 2:**
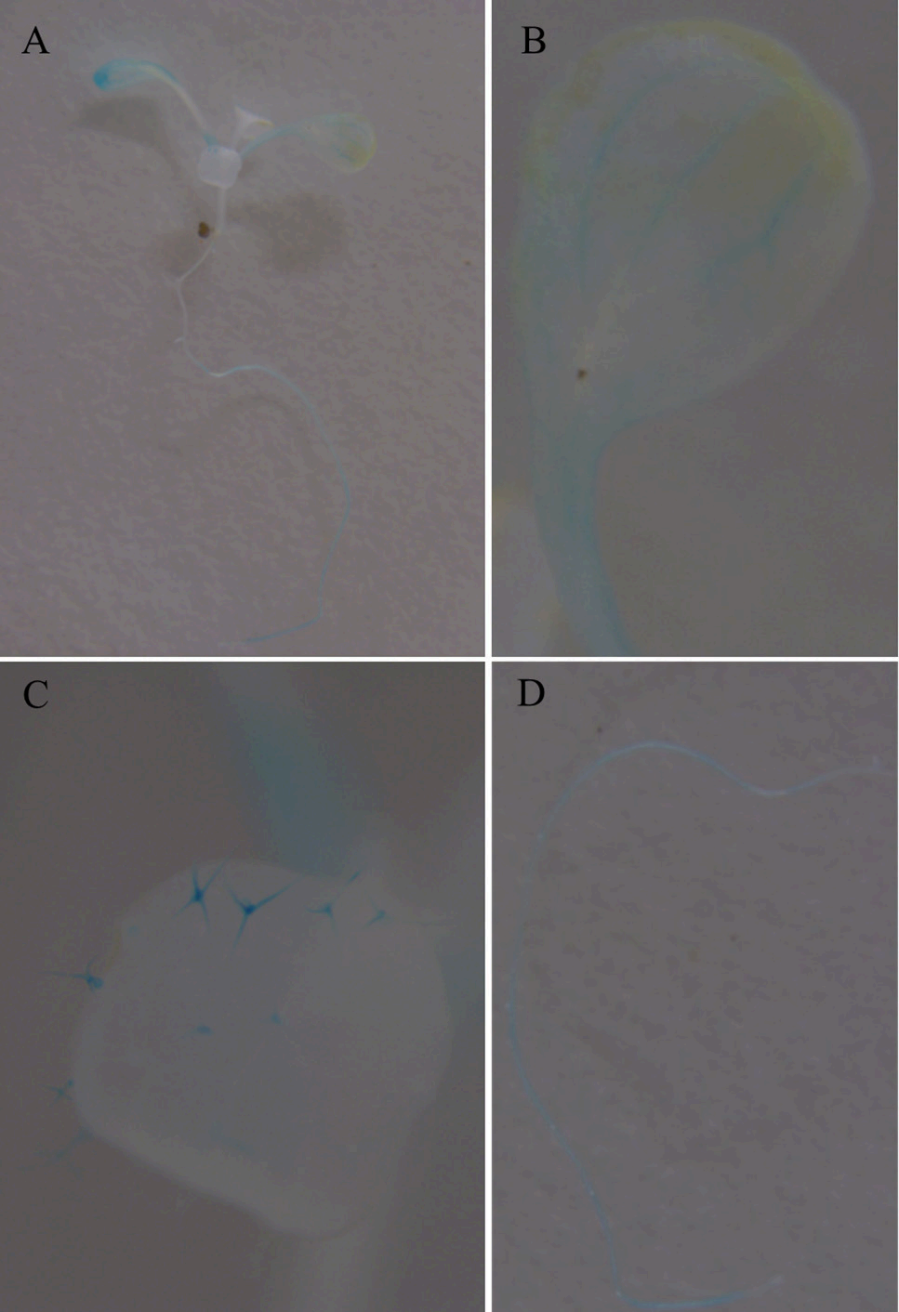
**GUS staining in 2-week-old Arabidopsis seedlings expressing ***ZjSGRpro::GUS:*** (A)** whole plant, **(B)** leaf. **(C)** Trichomes. **(D)** Root.

**Figure 3:**
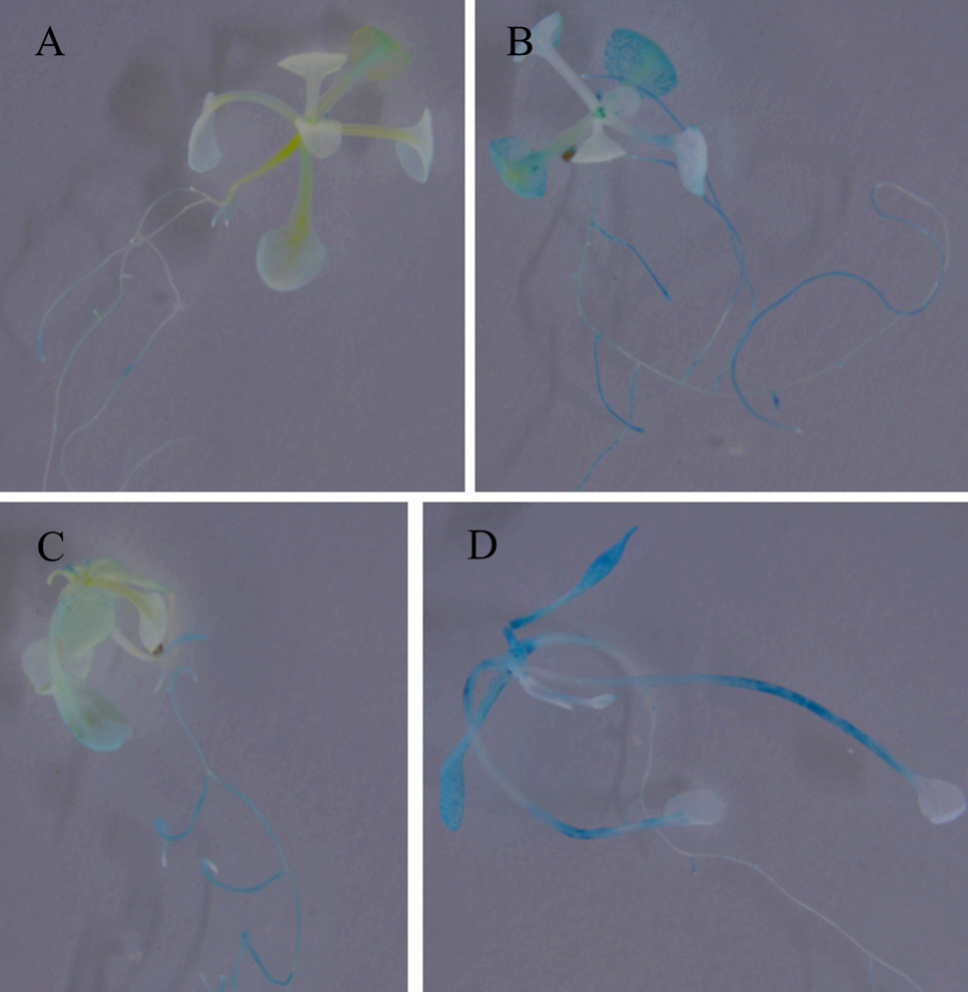
**GUS staining in 2-week-old Arabidopsis seedlings expressing ***ZjSGRpro::GUS*** induced by ABA, MeJA, and darkness for 3 days: (A)** Control, **(B)** 10 μM ABA, **(C)** 10 μM MeJA, **(D)** darkness.

To further investigate the expression characters of *ZjSGR*, qRT-PCR was carried out. It turns out that *ZjSGR* expressed most abundantly in zoysiagrass leaves (Figure 1A) and the transcript level was significantly up-regulated under dark treatment and natural senescence progress. In detail, *ZjSGR* expression level under dark and natural senescence inducements could be more than 11- and 245-folds higher than the initial levels, respectively (Figures 1B,C). Moreover, the results showed that ABA, and MeJA treatments could also induce *ZjSGR* expression levels to some degree, while SA suppressed the expression of *ZjSGR* during the 24 h-treatment period (Figures 1D–F). Histochemical analysis revealed that strong GUS activity was found in the leaves of stable transformed *ZjSGR*_*Pro*_*::GUS* Arabidopsis seedlings (Figure 2A). In detail, GUS signal was detected in the vein and petiole of the leaves (Figure 2B) and trichomes (Figure 2C), and also the region of roots (Figure 2D). After ABA, MeJA or dark inducement for 3 days, stronger GUS activity was detected (Figures 3B–D). The GUS assay results indicated that the *ZjSGR* could be induced by darkness, ABA, and MeJA treatments and it was in accordance with the qRT-PCR results.

### Overexpression of *ZjSGR* caused rapid yellowing phenotype in Arabidopsis

To investigate the function of *ZjSGR*, 35S::*ZjSGR:FLAG* plasmid was used to transform wild type (Col-0) Arabidopsis plants. In total, more than 40 independent transgenic lines were generated. Two representative T_3_ lines, SGR-3, andSGR-7, of transgenic lines with higher *ZjSGR* transcript levels were selected and used throughout the study. Western blot results showed that the target protein enriched in the *ZjSGR*-overexpressing transgenic Arabidopsis lines (Figure S1). The phenotype observation results showed that *ZjSGR* overexpression caused rapid yellowing phenotype in transgenic Arabidopsis plants (Figure 3A). In addition, overexpression of *ZjSGR* resulted in decreased biomass in transgenic Arabidopsis plants compared with control (Figure 3B). The result indicated that the 35S::*ZjSGR:FLAG* fusion fragment was sufficient to regulate chlorophyll degradation and it could be used as quantified materials for our future studies.

### *ZjSGR* could rescue the stay-green phenotype of *nye1-1* under dark inducement

To verify if *ZjSGR* could rescue the non-yellowing phenotype of the *nye1-1* (*stay-green* mutant), a 1850 bp fragment including the 5′ upstream sequence and the open reading frame was transformed into *nye1-1* Arabidopsis plants. More than 20 independent transgenic lines were generated in total and verified by PCR. Two representative T_3_ lines, Comp-14, and Comp-17, were selected based on the higher expression levels. Detached leaves were cultivated in the 3 mM MES buffer (pH 5.7) under total darkness conditions for 4 days. The results showed that the leaves of the complementary lines were turned to a yellowish color as the control (Col), while the *nye1-1* showed a stay-green phenotype (Figure 3C). Correspondingly, the expression level of *ZjSGR* in the comp-14 and comp-17 detached leaves were up-regulated significantly by darkness treatment (Figure 3D). The complement experiments proved the *ZjSGR* is a functional homology of *AtNYE1*, and could accelerate chlorophyll degradation.

### Overexpression of *ZjSGR* decreased chlorophyll content and impaired photosynthesis in Arabidopsis

**Figure 4:**
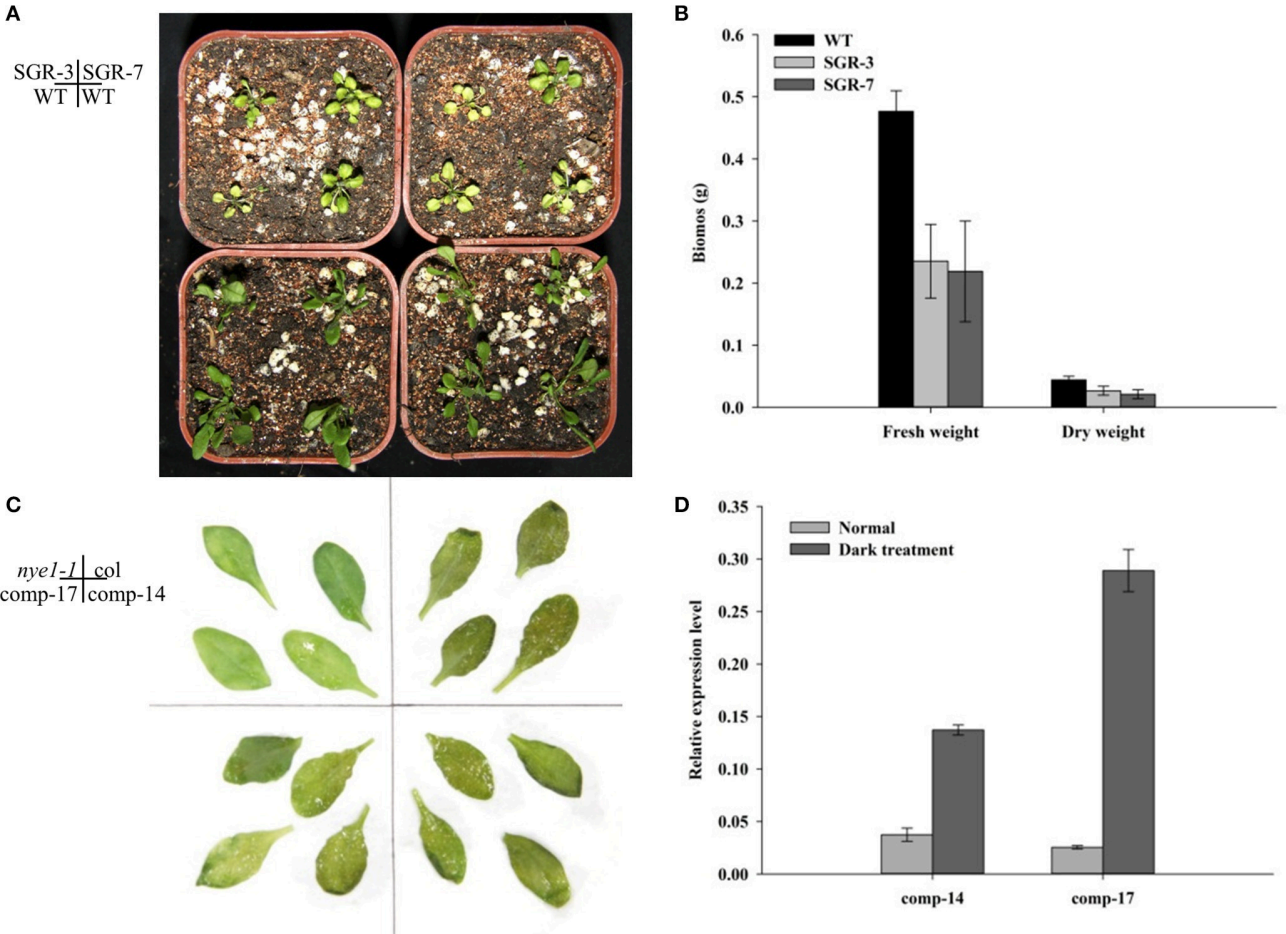
**Phenotype observation of ***ZjSGR*** overexpressing and complementary transgenic T3 lines. (A)** SGR-3 and SGR-7 overexpressing lines. **(B)** Biomass determination of SGR-3 and SGR-7. **(C)** comp-14 and comp-17 complementary lines. **(D)** The expression level of *ZjSGR* in the complementary lines.

**Figure 5:**
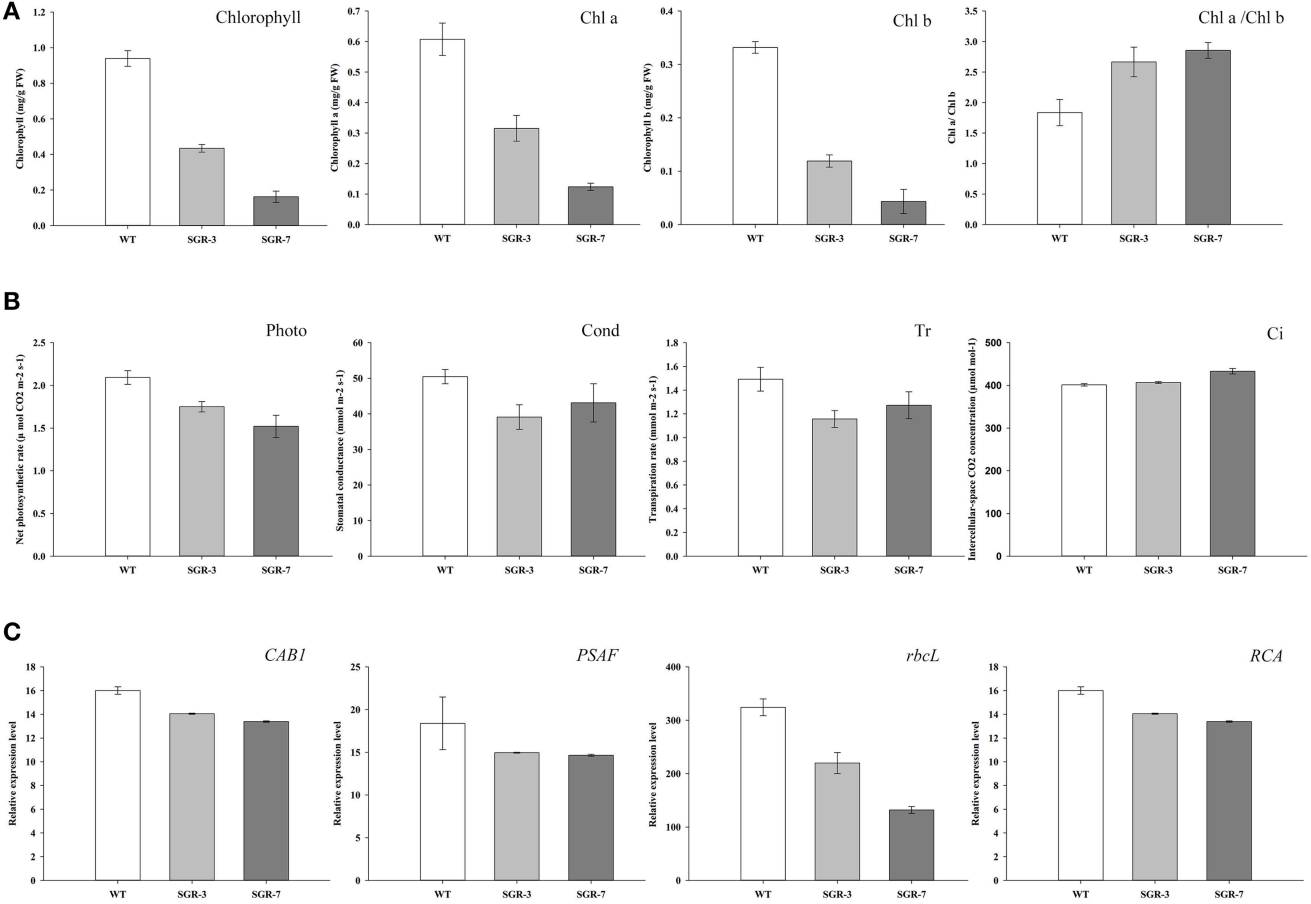
**Determination of chlorophyll and photosynthesis in ***ZjSGR***-overexpressing Arabidopsis: (A)** Chlorophyll contents, **(B)** Photosynthesis ability. Photo, the net photosynthetic rate; Cond, stomatal conductance; Ci, intercellular-space CO2 concentration; and Tr, transpiration rate. **(C)** Expression levels of photosynthetic related genes. *CAB1*, chlorophyll a/b binding gene; *PSAF*, the photosystem I component encoding gene; *rbcL*, the Rubisco large submit gene; *RCA*, the Rubisco activase gene.

During the phenotype observation, a general rapid yellowing character was found in both the *ZjSGR*-overexpressing and also the complementary transgenic lines (Figure 3). To confirm the roles of *ZjSGR* in chlorophyll physiological metabolism, total chlorophyll, chlorophyll a and chlorophyll b contents were measured in both *ZjSGR*-overexpressing and WT Arabidopsis plants. As shown in **Figure 5A**, total chlorophyll content, chlorophyll a, and chlorophyll b contents were much lower in the representative *ZjSGR*-overexpressing lines than the control. This is consisted with the yellowing phenotype of the transgenic Arabidopsis plants (Figure 4). Moreover, the ratio of Chl a/b in transgenic plants were higher than the WT plants, significantly.

Further analysis of the photosynthetic system revealed that the net photosynthetic rate, stomatal conductance and transpiration rate were lower in the transgenic plants than the control, whereas the intercellular-space CO_2_ concentration was slightly higher under normal growth conditions (Figure 5B). We then focused on the underlying genetic mechanisms by examining several photosynthesis-related genes in both *ZjSGR*-overexpressing and WT plants. As shown in Figure 5C, the expression level of the *CAB1* (chlorophyll a/b binding gene), *PSAF* (the photosystem I component encoding gene), *rbcL* (the Rubisco large submit gene), and *RCA* (the Rubisco activase gene) were all found significantly lower in the transgenic plants than the control. It indicated that, overexpression of *ZjSGR* caused decreased chlorophyll content and photosynthetic system efficiency.

### ZjSGR is located in the chloroplast and could impair chloroplast structure in transgenic plants

**Figure 6:**
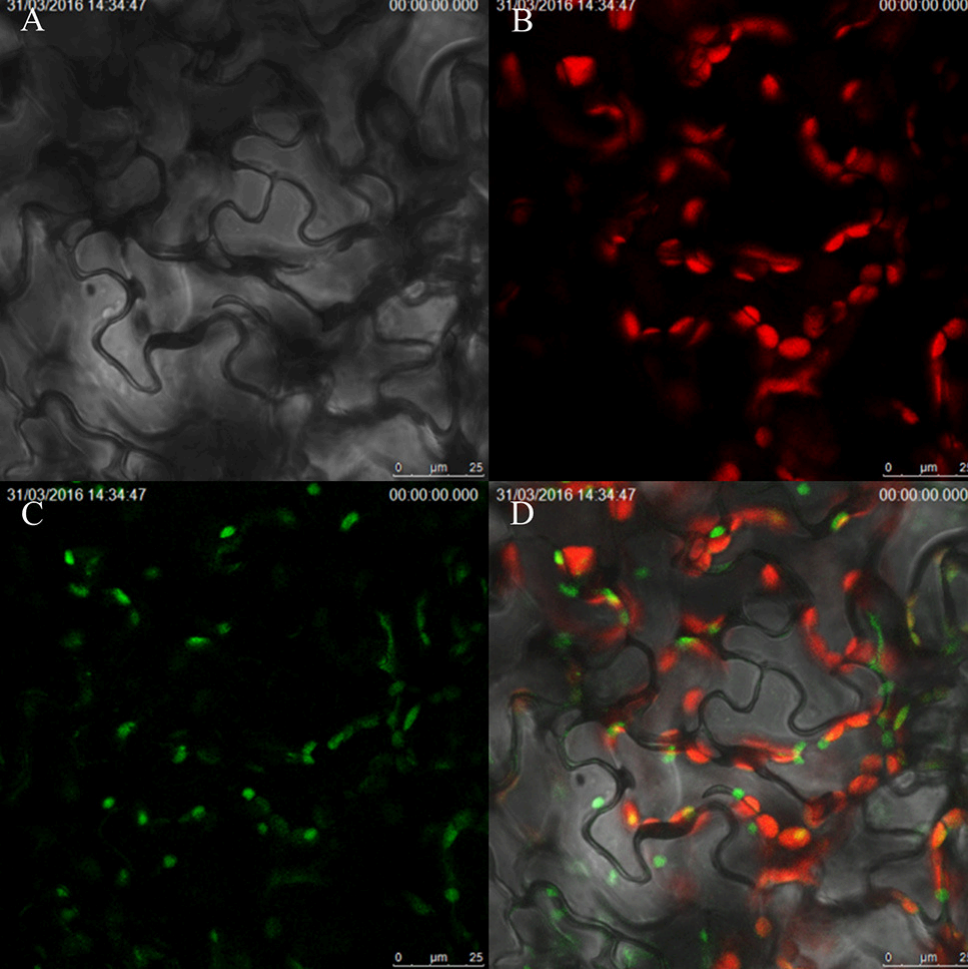
**Subcellular localization of ZjSGR in stable transformed Arabidopsis: (A)** bright field, **(B)** chloroplast auto-fluorescence, **(C)** 35S::*ZjSGR:YFP*, **(D)** merged field.

To explore the subcellular localization pattern of ZjSGR, stable transformed 35S::*ZjSGR:YFP* Arabidopsis plants leaves were observed using laser confocal scanning microscope. It turned out that the ZjSGR protein was localized in the chloroplasts obviously (Figure 6). However, after observing dozens of slides, a general phenomenon drew our attention that the strong YFP signal were only found in the area where exhibited week chloroplast auto-fluorescence. It led to the hypothesis that overexpression of *ZjSGR* might impair the chloroplast structure.

**Figure 7:**
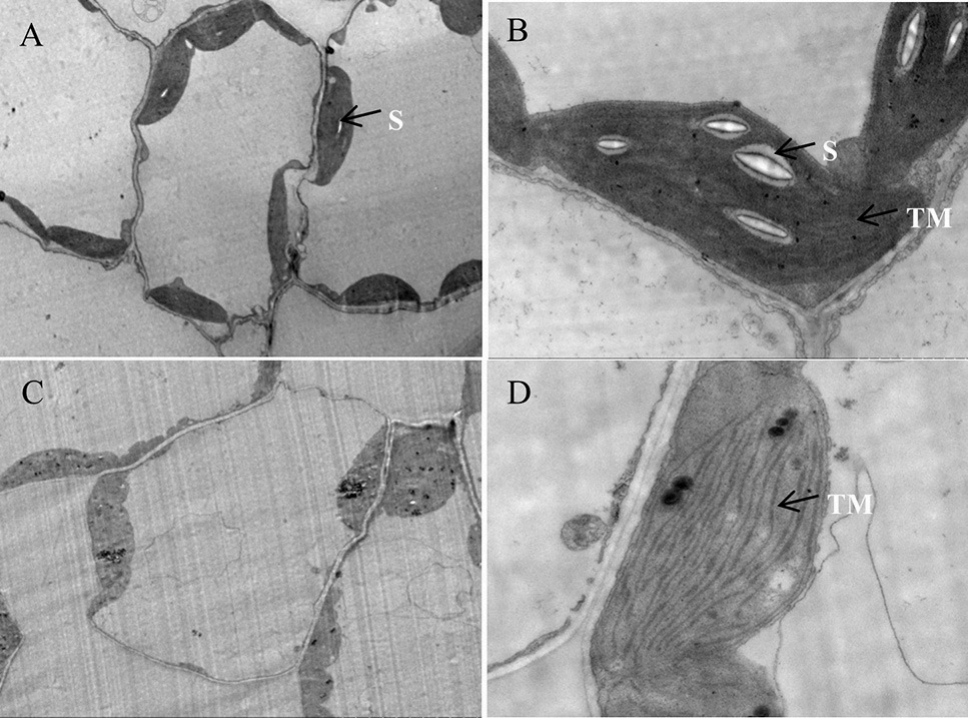
**Ultrastructure of chloroplasts in (A,B)** wild type, and **(C,D)** *ZjSGR*-overexpressing line. S, starch granule; TM, thylakoid membrane.

To verify our assumption, the ultrastructure of chloroplasts in both the transient (tobacco) and stable transformed (Arabidopsis) *ZjSGR*-overexpressing plants were observed using TEM. The results showed that transient overexpression of *ZjSGR* caused drastic decomposition of chloroplasts in tobacco leaves, corresponding with the interesting subcellular localization pattern (Figure S2). We then focused on the stable transformed Arabidopsis plants with fast yellowing phenotype. The structure of the chloroplasts in WT plants under normal growth conditions maintained a high stability status and didn't show degeneration tendency (Figures 7A,B). However, it exhibited a degeneration of chloroplast structure of the fast yellowing leaves overexpressing *ZjSGR* under normal growth condition, in which there were fewer starch granule and loose thylakoid membrane (Figures 7C,D). Taken together, these results indicate that there was drastic chloroplast decomposition progress in the *ZjSGR*-overexpressing leaves which might in turn result in the decreased photosynthesis as well as the interesting subcellular localization character.

### Global expression analysis revealed that *ZjSGR*-overexpression accelerated the precocious senescence in developing leaves

In this study, the global gene expression analysis was performed with RNA sequencing (RNA-seq) using *ZjSGR*-overexpressing Arabidopsis and with WT as control to explore the molecular network of *ZjSGR* in senescence. Pearson's correlation coefficient was employed to test the reliability of RNA-seq data (Schulze et al., 2012). Correlation coefficients (approximately close to 1) suggested a strong correlation among the biological replicates in WT and *ZjSGR*-overexpressing plants (Figure S3). Five hundred and ninety five assembled transcripts in total were significantly differently expressed (log_2_ ≥ 1, *q* < 0.05; Table S3), of which 499 (83.87%) were up-regulated, whereas 96 (16.13%) down-regulated (Figure S4). To verify the digital expression level of RNA-seq data, qRT-PCR was carried out by investigating the expression levels of 10 representative genes. As shown in Table 2, the qRT-PCR data were generally in accordance with the digital expression levels, confirming that the RNA-seq data were faithful enough for next step analysis.

**Table 2:** **Verification of DEGs in ***ZjSGR*** overexpression transgenic and wild type Arabidopsis plants by qRT-PCR analysis**.

**Gene**	**#ID**	**SGR/WT (log_2_ FC)**	
		**Digital expression**	**qRT-PCR**
SEN1	AT4G35770	2.34	2.61
SAG21	AT4G02380	1.24	1.43
SAG14	AT5G20230	3.21	3.37
NYC1	AT4G13250	1.20	1.06
YUC9	AT1G04180	−3.83	−5.03
IAA6	AT1G52830	−2.13	−2.38
IAA29	AT4G32280	−3.28	−3.71
NAC29	AT1G69490	1.84	2.55
WRKY6	AT1G62300	1.88	2.94
NAC47	AT3G04070	2.33	2.94

**Figure 8:**
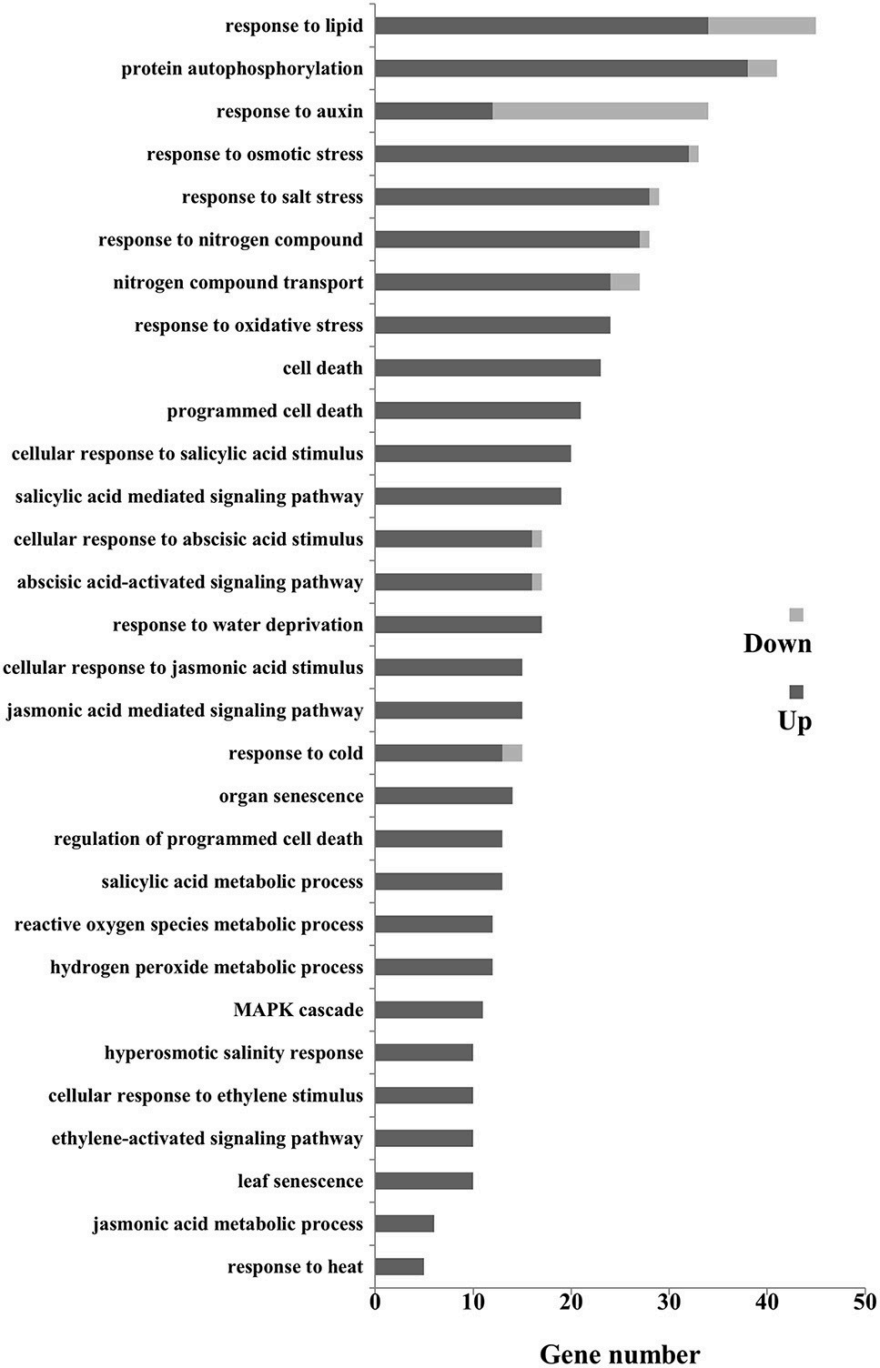
**Gene ontology (Go) classification for senescence related DEGs in WT and ***ZjSGR***-overexpressing Arabidopsis**. Only the biological processes were used for GO term analysis.

The different expressed genes (DEGs) could be classified into 43 groups (Figure S5) according to the allocated gene ontology (GO) terms (Table S4). Gene Ontology analysis revealed that catalytic activity, binding, and nucleic acid binding transcription factor activity were most overrepresented among the molecular functions; cell part, cell, and organelle were most overrepresented among the cellular component. Further classification in terms of the biological process identified numerous senescence-related genes including nitrogen compound transport, programmed cell death, organ senescence, leaf senescence, phytohormone (auxin, ABA, ET, JA, and SA), and those participated in defense responses (Figure 8). We then focused on the senescence markers of the RNA-seq data. The transcriptional amounts of the 10 putative leaf senescence-associated genes were all up-regulated in *ZjSGR*-overexpressing plants.

Additionally, all the DEGs were analyzed to identify the metabolic pathways using the KOBAS system. In total, 78 KEGG pathways were identified and among which the plant hormone signal transduction pathway was significantly enriched (Corrected *P* < 0.05). To gain insight into the hormone-mediated regulation in senescence, we mapped the DEGs to the Arabidopsis Hormone Database. The results showed that 176 of the 595 DEGs were matched to the SA, ABA, Auxin, JA, and ET associated processes, including response, signaling, biosynthesis and metabolism (Table 3). The largest group was related to SA, and only 2 of the 42 DEGs were down-regulated. In addition, there were only two, one or none down-regulated DEGs found in the ABA, JA, and Ethylene related transcriptional regulations, respectively. Of particular interests, 38 auxin signaling and responsive DEGs was enriched but only 14 of them were up-regulated.

**Table 3:** **Hormone-related genes differentially expressed in ***ZjSGR***-overexpressing Arabidopsis leaves**.

**Hormones**	**Total number**	**Up-regulated**	**Down-regulated**
SA	42	40	2
Auxin	38	14	24
ABA	35	33	2
JA	32	31	1
Ethylene	29	29	0

Interestingly, 3.70% (22 of 595) of up- or down- regulated genes encode chloroplast-related proteins (Table S5) in our study. In addition, 54 TF (transcription factor) transcripts from 20 TF families were identified among the 595 DEGs (Table S6). The WRKY, NAC, AP2-EREBP, and MYB families ranked the top four largest families in the RNA-seq data (Table 4). The NAC, WRKY and AP2-EREBP TFs were up-regulated significantly, whereas the AUX/IAA TFs were down-regulated in our study.

**Table 4:** **Abiotic stress response-related DEGs in wild type control and ***ZjSGR***-overexpressing transgenic plants**.

**Gene family**	**ID**	**log_2_FC**	**padj**	**Annotation**
NAC	AT1G02220	1.8826	0.0020227	NAC domain-containing protein 3
	AT1G02230	1.7398	0.0052586	NAC domain-containing protein 4
	AT3G04070	2.3338	0.00083176	NAC domain-containing protein 10
	AT1G69490	1.8425	0.000561	NAC transcription factor 29
	AT5G08790	1.5415	0.035491	NAC transcription factor 81
	AT5G39610	1.3207	0.00018779	NAC transcription factor 92
	AT3G04060	2.1199	0.0025559	NAC transcription factor 100
WRKY	AT1G62300	1.8769	0.0018992	WRKY transcription factor 6
	AT5G46350	1.7111	0.012848	Probable WRKY transcription factor 8
	AT2G23320	1.3137	0.038549	Probable WRKY transcription factor 15
	AT2G30250	1.1619	0.035656	Probable WRKY transcription factor 25
	AT5G07100	1.4654	5.73E-07	Probable WRKY transcription factor 26
	AT3G01970	1.7049	3.39E-09	Probable WRKY transcription factor 45
	AT5G64810	1.9589	0.00098186	Probable WRKY transcription factor 51
	AT2G40750	1.3998	2.93E-05	Probable WRKY transcription factor 54
	AT3G01080	1.6474	0.00039729	Probable WRKY transcription factor 58
	AT1G18860	2.0537	0.032078	Probable WRKY transcription factor 61
	AT1G66600	2.1873	0.02429	Probable WRKY transcription factor 63
	AT3G56400	1.9338	0.00016706	Probable WRKY transcription factor 70
AP2-EREBP	AT1G75490	1.3706	0.00018407	Dehydration-responsive element-binding protein 2D
	AT3G23240	1.7056	0.0010819	Ethylene-responsive transcription factor 1B
	AT3G50260	2.1082	0.0071382	Ethylene-responsive transcription factor ERF011
	AT4G17500	2.2825	0.00030021	Ethylene-responsive transcription factor 1A
	AT5G47220	1.1646	0.0002348	Ethylene-responsive transcription factor 2
	AT1G25560	1.0054	0.039972	AP2/ERF and B3 domain-containing transcription repressor TEM1
MYB	AT1G71030	1.3229	0.011338	MYB transcription factor 3
	AT4G05100	2.4321	0.027624	MYB transcription factor 39
	AT4G21440	2.4943	0.016251	MYB transcription factor 39
	AT1G48000	2.1311	0.012472	MYB transcription factor 108
	AT2G18328	−1.2388	0.011778	Protein RADIALIS-like 4
	AT4G36570	−1.2784	0.022458	Protein RADIALIS-like 3
	AT5G08520	−1.1277	0.0004582	Transcription factor DIVARICATA
	AT1G01520	2.0641	0.0017054	Protein REVEILLE 3
bHLH	AT2G18300	−1.2332	2.32E-05	Transcription factor HBI1
	AT1G02340	−1.6919	0.039972	Transcription factor HFR1
bZIP	AT2G42380	−1.4225	1.30E-05	Basic leucine zipper 34
	AT2G46270	1.5046	0.030884	G-box-binding factor 3
AUX/IAA transcriptional regulator	AT1G04240	−1.2816	0.013065	Auxin-responsive protein IAA 3
	AT1G52830	−2.1286	0.0036074	Auxin-responsive protein IAA6
	AT4G32280	−3.2776	1.10E-05	Auxin-responsive protein IAA29

*Significant differences were determined with FDR < 0.05 and absolute value of log_2_ FC ≥ 1*.

## Discussion

The identification of genes that play important roles in controlling the chlorophyll degradation and manipulating senescence progresses provided new access to study plant senescence at molecular level. Herein, a stay-green protein gene was identified from *Z. japonica*. ZjSGR showed high homologous with the stay-green proteins from other species, indicating its potential roles in manipulating chlorophyll degradation and senescent progresses. The *cis*-element prediction in the promoter indicated that the expression of *ZjSGR* might be induced by related environmental conditions and hormones. The qRT-PCR results proved our assumption that the expression level of Z*jSGR* is in positive correlation with senescent stages. It implied that *ZjSGR* could be used as an ideal senescence related marker gene. Both the GUS assay and the qRT-PCR results proved that *ZjSGR* could be induced by dark treatment, and *ZjSGR* expressed abundantly in the petiole, trichomes, and veins of leaves, suggesting potential roles for *ZjSGR* in different leaf structures and developments. In addition, the expression of *ZjSGR* could be up-regulated by ABA and MeJA, and down-regulated by SA application, providing a potential to regulate its transcript through effective management means.

Overexpression and complementation test results showed that overexpression of *ZjSGR* accelerated chlorophyll degradation and *ZjSGR* could rescue the stay-green phenotype of *nye1-1*, suggesting that *ZjSGR* is a functional homolog of *AtNYE1* from Arabidopsis. The total chlorophyll content was decreased in the *ZjSGR*-overexpressing plants and that was in accordant with the rapid yellowing phenotype. It has been considered that the first step in chlorophyll degradation is the conversion of Chl b–Chl a (Hörtensteiner, 2009). The increased Chl a/b ratio in transgenic Arabidopsis was consisted with the yellowing phenotype in our study. Moreover, overexpression of *ZjSGR* results in the decreased net photosynthetic rate, stomatal conductance, and transpiration rate, indicating that *ZjSGR*-overexpression impaired photosynthetic system. The slight higher intercellular CO_2_ concentration also suggested that other photochemical or biochemical activity were probably impaired in transgenic plants (Guo et al., 2004). We further investigated several photosynthesis related genes, including *CAB1, PSAF, rbcL*, and *RCA*. The expression levels of all the investigated genes were declined in transgenic plants compared with control, implying the possible involvement of *ZjSGR* in modulating photosynthetic gene expression directly or indirectly. Taken together, the results proved that overexpression of *ZjSGR* could accelerate chlorophyll degradation and impair photosynthesis in transgenic Arabidopsis through genetic and biochemical regulation.

The subcellular localization determination proved that ZjSGR was a chloroplast localized protein. This is consistent with the homologous stay-green proteins in *A. thaliana* (Ren et al., 2007), *O. sativa* (Park et al., 2007), and *M. truncatula* (Zhou et al., 2011). TEM observation proved that overexpression of *ZjSGR* could impair the chloroplasts structure in transgenic Arabidopsis. Previous studies investigated the chloroplast structure in the *stay-green* mutants under dark inducements, and the results showed that the chloroplast decomposed much slower in the *stay-green* mutants than the control (Park et al., 2007; Zhou et al., 2011). In this study, the interesting subcellular localization pattern inspired us to determine if *ZjSGR* overexpression could impair the ultrastructure of chloroplasts. The relative fewer starch granules and loose thylakoid membrane indicated that the *ZjSGR* accelerate the decomposition progress in *ZjSGR*-overexpressing plants under normal growth conditions. Ultrastructural studies proved that chloroplasts are the first organelle to be decomposed in leaf senescence progress (Dodge, 1970), and resulting in leaf lipids and proteins to be recycled in next step metabolism (Ischebeck et al., 2006). Our results suggested that *ZjSGR* accelerated chloroplast decomposition and might in turn result in the regulation of downstream metabolism.

A large number of genes are reported differently expressed in the onset and progression of senescence and several networks of gene regulation have been proposed in recent years (Schippers et al., 2015). The RNA-seq data allowed us to further explore the function of *ZjSGR* from a broader perspective. It revealed that 499 up- and 96 down-regulated genes in total were identified in the *ZjSGR*-overexpressing plants, implying a preferential role of *ZjSGR* in regulating gene expression. *SEN1, NYC1, SAG21, SAG14*, and *SAG39* were selected as positive markers to monitor the senescence progress in plants (Jing et al., 2002; Ren et al., 2010; Zhou et al., 2011; Jibran et al., 2015; Salleh et al., 2016). The expression levels of all the senescence related DEGs in *ZjSGR*-overexpressing plants were proved to be up-regulated, implying that the accelerated senescence progress in the transgenic plants. The GO analysis results showed that *ZjSGR* affected the transcripts of a large portion of genes involved in biotic and abiotic responses in a direct or indirect way, supporting the view that plant defense and senescence extensively co-regulate many genes in these responses (Schenk et al., 2005). The genetic and biochemical regulatory mechanisms of SGR in chlorophyll degradation are largely elusive to date (Zhou et al., 2011; Luo et al., 2013; Sakuraba et al., 2015). In this study, the RNA-seq data revealed that only 3.70% of the DEGs are chloroplast-related genes. It suggests that, rather than directly regulate specific component of the chlorophyll catabolism pathway, *ZjSGR* may play multi-roles beyond chlorophyll degradation. Because ZjSGR is localized to the chloroplasts, it is possible that the changes in transcript levels resulted from the consequences of secondary effects in chloroplasts decomposition. This result is similar to the microarray analysis of *M. truncatula stay-green* mutants that proved the extended roles of MtSGR beyond chlorophyll degradation (Zhou et al., 2011).

Senescence involves a rather complex network of hormone signal transduction, such as ABA, MeJA, and Auxin mediated pathways. In our study, KEGG analysis was utilized to explore the metabolic pathways in senescence. The significantly enriched hormone signal transduction pathway suggested its potential roles in senescence. Auxin has been reported to function as a senescence retardant (Mueller-Roeber and Balazadeh, 2014), but its mode of action with respect to senescence is only vaguely defined compared with other hormones such as ABA, MeJA, and GA. Koyama et al. (2013) reported that the transcripts for *IAA6* and *IAA29* were reduced in precocious leaf senescence. *SAUR* family genes have been widely used as auxin inducible reporters, and the expression could be induced by auxin rapidly and strongly both *in vitro* and *in vivo* (Hou et al., 2012; Wu et al., 2012). The RNA-seq results suggested a decreasing trend of auxin response at the transcription level. ABA, Ethylene and JA play important roles in manipulating senescence and environmental stresses in plants (Schippers et al., 2015). The data showed that *HAB1* and *SNRK2-9* were up-regulated in ABA signal transduction, reflecting a possible negative feedback regulation of ABA metabolism (Wang, 2015), and ABA mediate pathway for stress response in *ZjSGR*-overexpressing plants (Fujii et al., 2011). This is consistent with our previous study that overexpression of *SGR* drastically increased the ABA contents in tobacco (Teng et al., 2015). *ERF1* and *ERF2* are biosynthetic and responsive factors involving in ethylene pathway (Tsai et al., 2014). *JAZ9*, one member of TIFY family genes, fine-tunes the expression levels of JA-responsive genes in plant stress (Wu et al., 2014). The transcriptional increases of *ERF1* and *ERF2*, and *JAZ9* reflected activated ethylene and JA signal transduction contributing to leaf senescence, respectively.

Transcription factors (TFs), by binding to distinct *cis*-regulatory elements to regulate gene expression, have been proved to be central elements in the senescence networks (Balazadeh et al., 2008). NAC TFs have been reported to function efficiently in chlorophyll degradation and leaf senescence, and actively participant in ABA, MeJA, and ethylene signaling which could act back on leaf senescence (Bu et al., 2008; Yang et al., 2011; Kim et al., 2014; Sakuraba et al., 2015; Takasaki et al., 2015; Li et al., 2016). Take the significantly up-regulated *AtNAC92* as an example in our study, it was reported to work together with other NAC domain proteins to regulate the expression of many genes in senescence (Balazadeh et al., 2010; Breeze and Buchanan-Wollaston, 2011). *AtWRKY6* provides the first evidence that supporting specific WRKY proteins regulate senescence in Arabidopsis (Robatzek and Somssich, 2001). Since then, more and more functional genomic and global transcript studies proved that WRKY TFs could transcriptionally regulate plant senescing processes (Balazadeh et al., 2008; Rinerson et al., 2015). By cross-talking with others, AP2-EREBP TFs are likely to regulate leaf senescence-associated signaling pathways including ROS, ethylene, ABA, and JA (Mizoi et al., 2012). Additionally, MYB TFs were reported to work in plant senescence and various kinds of environmental stresses (Zhang et al., 2011). In this study, the activated senescence in *ZjSGR*-overexpressing plants may be due, in part, to the up-regulated expression levels of the NAC, WRKY and AP2-EREBP TFs (Koyama, 2014). The global profiling data provides informative data for further study in exploring the molecular regulatory mechanism of *SGR* and represents an invaluable resource for further investigate senescence processes in plant.

## Conclusions

In summary, a stay-green protein gene, *ZjSGR*, was identified in this study. It could be up-regulated in darkness and natural senescence processes, as well as ABA and MeJA inducements. Functional analysis proved that overexpression of *ZjSGR* caused rapid yellowing phenotype and could rescue the stay-green phenotype of *nye1-1*. The chloroplasts localized ZjSGR protein could impair the chloroplasts structure, and decrease chlorophyll content and impaired photosynthesis in Arabidopsis. The functional analysis accompanied with the RNA-seq data proved *ZjSGR* could play important roles in chlorophyll degradation and senescence progress. This is closely related to the hormone signal transduction and the regulation of a large number of senescence and environmental stress related genes. Our study provides a better understanding of the roles of SGRs, and new insight into the senescence and chlorophyll degradation mechanisms in plants.

## Author contributions

KT and YC conceived the study and designed the experiments. KT performed the experiment, and analysed the data with suggestions by ZC, XL, XS, XL, LX, and LH. KT wrote the manuscript.

### Conflict of interest statement

The authors declare that the research was conducted in the absence of any commercial or financial relationships that could be construed as a potential conflict of interest.

## Supplementary material

Figure S1 **Western blot analysis of ***ZjSGR***-overexpressing Arabidopsis lines**.

Figure S2 **Chloroplast structure in (A,B)** WT (infiltrated with recombinant agrobacteria as control) and **(C,D)** transient *ZjSGR*-overexpressing tobacco leaves (infiltrated with recombinant agrobacteria containing *35S::ZjSGR* plasmid).

Figure S3 **Pearson correlation between samples**.

Figure S4 **Volcano plot of the DEGs**.

Figure S5 **Gene ontology (GO) classification of the DEGs**.

Table S1 **Primers used for the RNA-seq data verification**.

Table S2 *****Cis***-regulatory elements of the 5′ up-stream sequence of ***ZjSGR*****.

Table S3 **List of the assembled differently expressed transcripts (absolute value of log_**2**_ FC ≥ 1, and ***q*** < 0.05) in ***ZjSGR*** transgenic Arabidopsis compared with WT**.

Table S4 **GO analysis list**.

Table S5 **Up- or down- regulated genes encoding chloroplast-related proteins**.

Table S6 **TFs analysis lists**.

## Abbreviations

qRT-PCR: quantitative real-time PCR
ABA: abscisic acid
MeJA: methyl jasmonate
ET: ethylene
SA: salicylic acid
GUS: β-Glucuronidase
TEM: transmission electron microscopy
RNA-seq: RNA sequencing
DEGs: differently expressed genes
GO: Gene Ontology
KEGG: Kyoto Encyclopedia of Genes and Genomes
TFs: transcript factors.
