# Supplementary figures and images for: Functional and RNA-Sequencing Analysis Revealed Expression of a Novel Stay-Green Gene from Zoysia japonica (ZjSGR) Caused Chlorophyll Degradation and Accelerated Senescence in Arabidopsis
Source: Front Plant Sci. 2016 Dec 16;7:1894. doi: 10.3389/fpls.2016.01894 (PMC5159421; doi:10.3389/fpls.2016.01894)

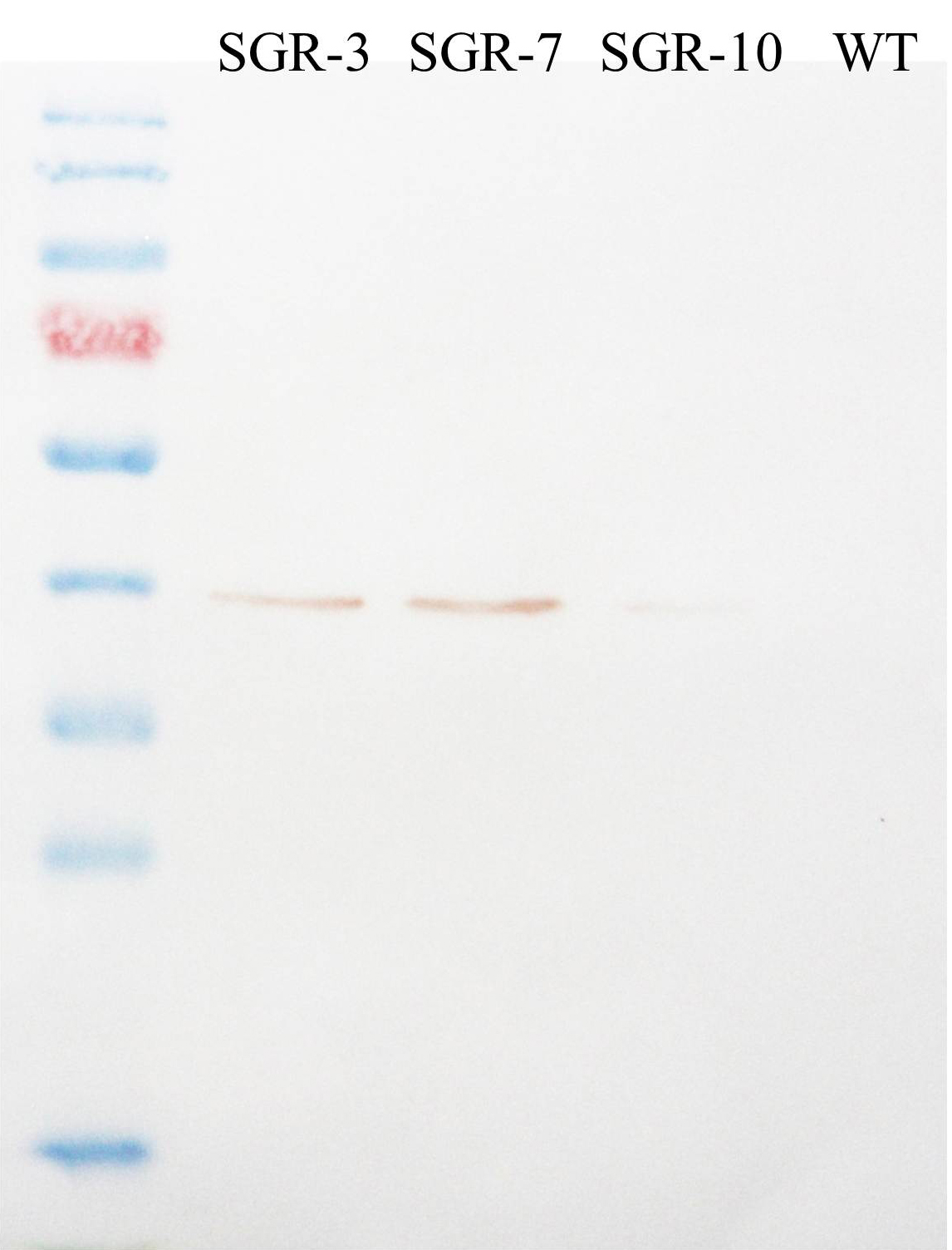

Supplement: Figure S1 — Western blot analysis of ZjSGR-overexpressing Arabidopsis lines. [file Image1.JPEG]

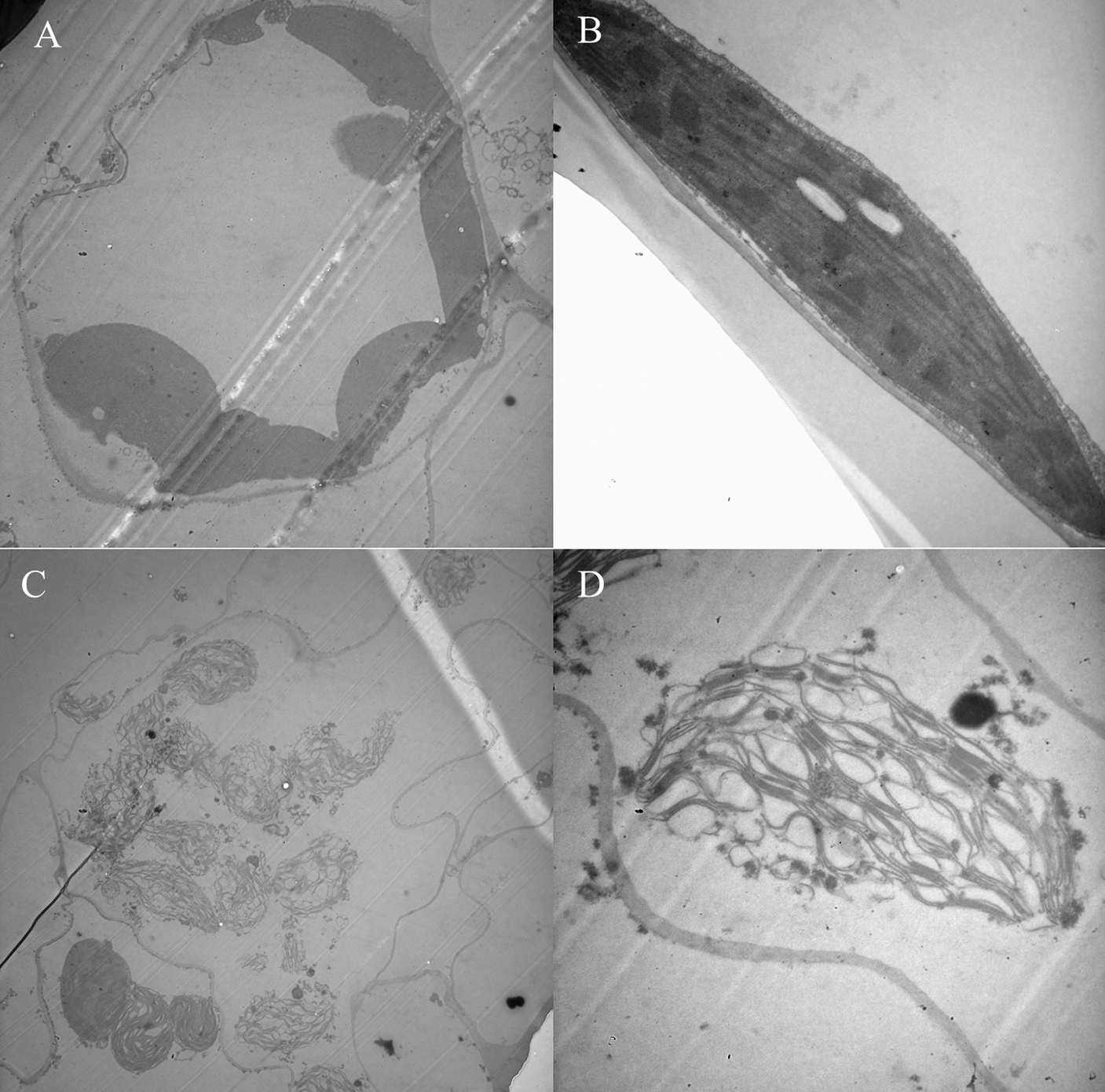

Supplement: Figure S2 — Chloroplast structure in (A,B) WT (infiltrated with recombinant agrobacteria as control) and (C,D) transient ZjSGR-overexpressing tobacco leaves (infiltrated with recombinant agrobacteria containing 35S::ZjSGR plasmid). [file Image2.TIF]

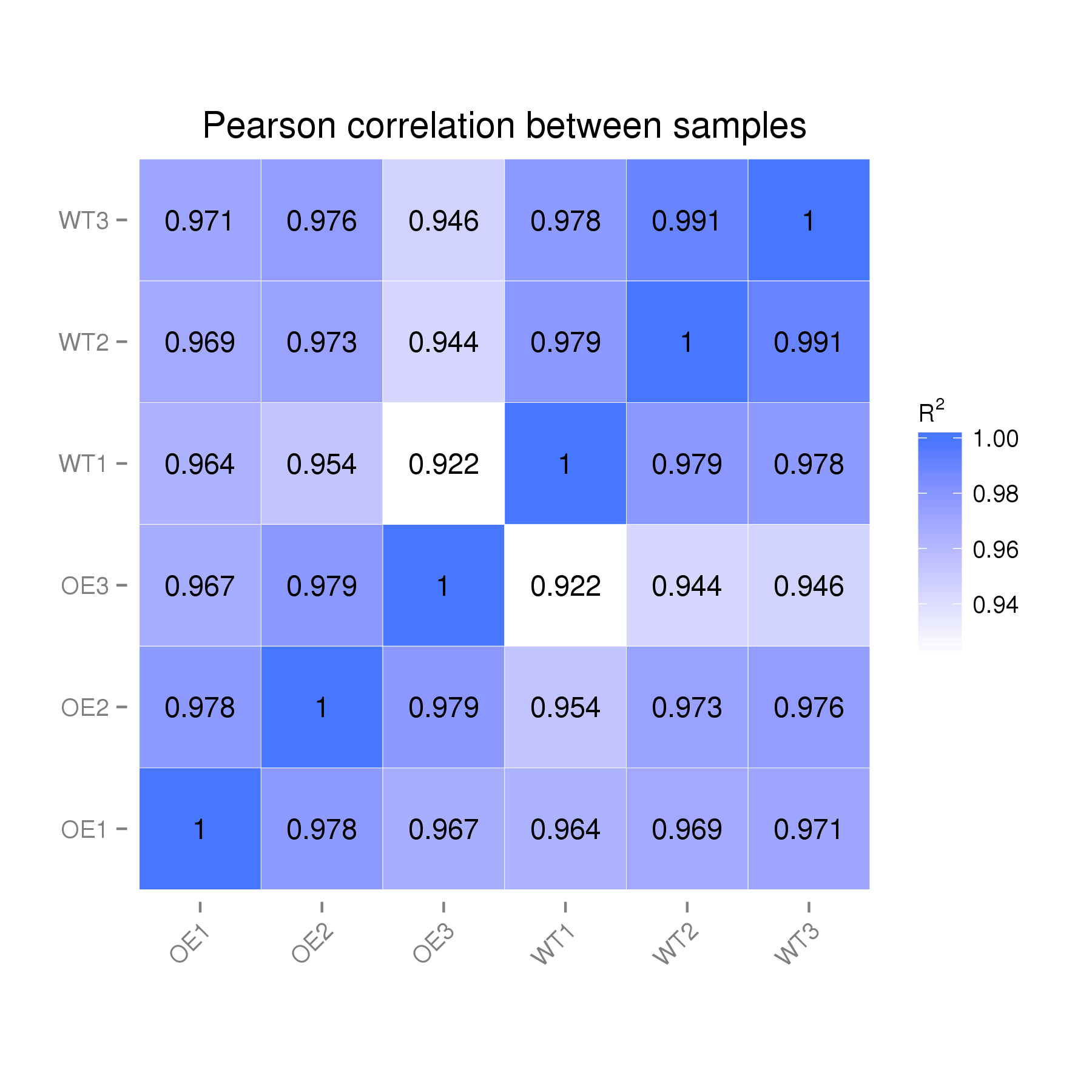

Supplement: Figure S3 — Pearson correlation between samples. [file Image3.PNG]

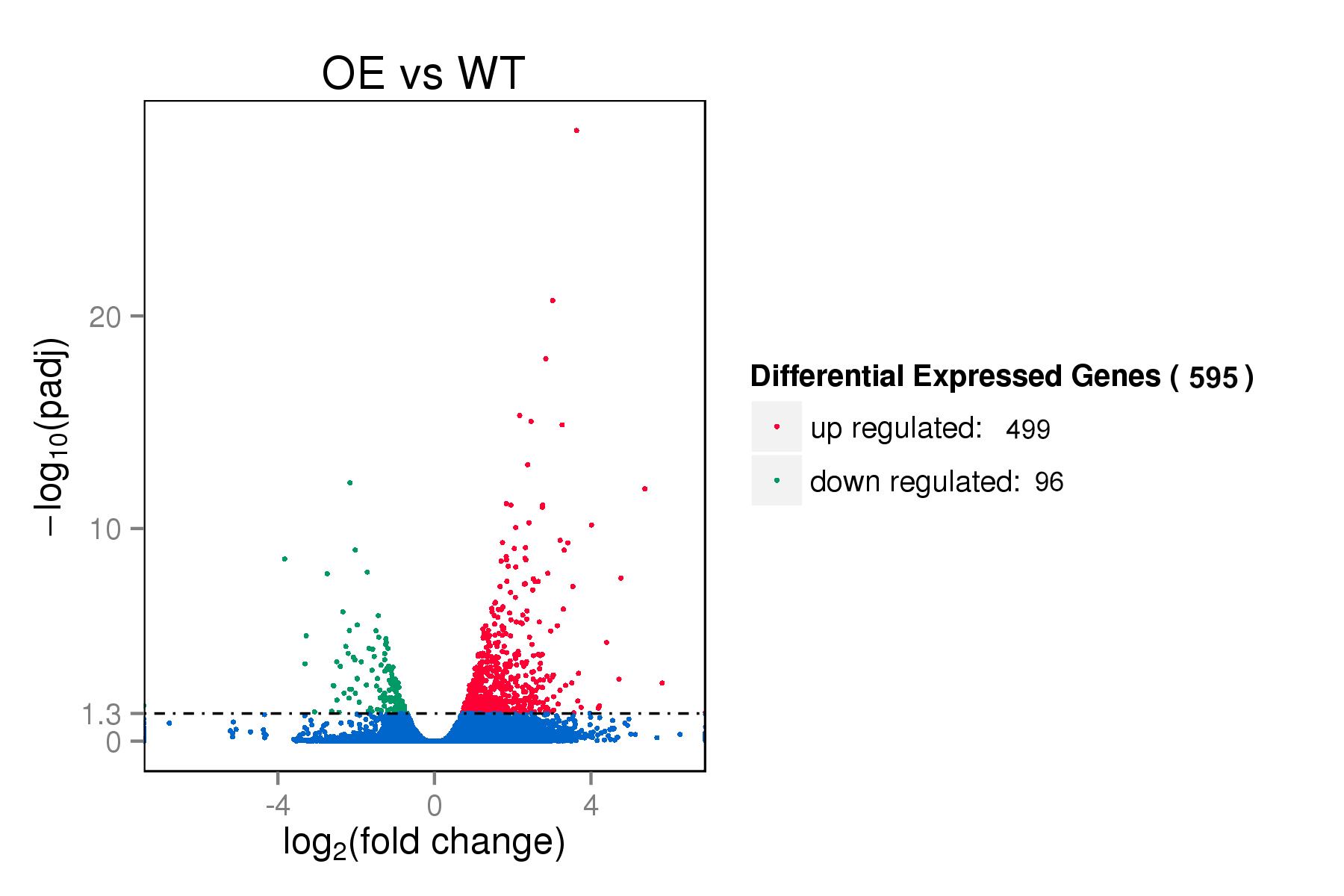

Supplement: Figure S4 — Volcano plot of the DEGs. [file Image4.JPEG]

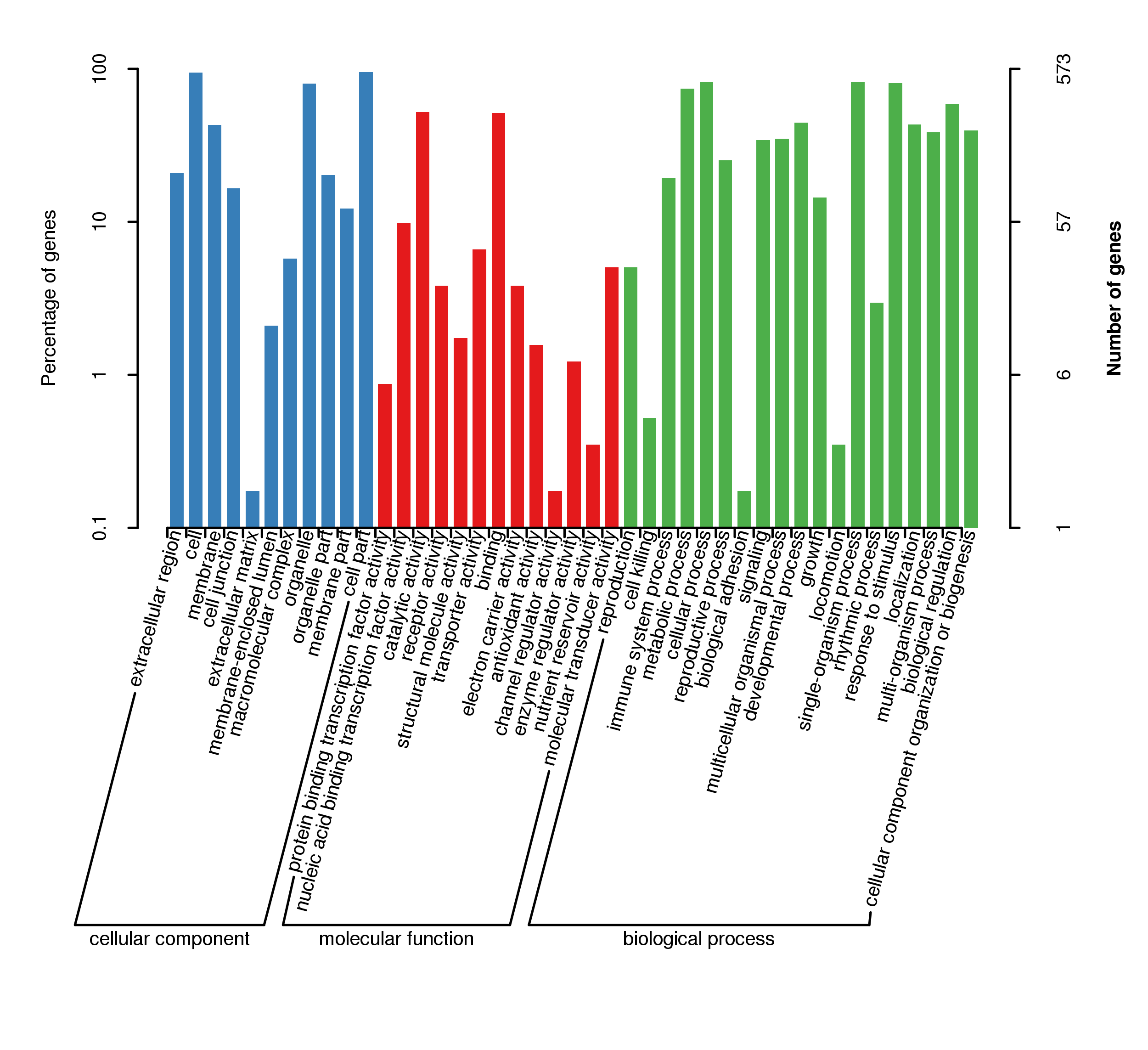

Supplement: Figure S5 — Gene ontology (GO) classification of the DEGs. [file Image5.PNG]
