# Supplementary material for: Functional and RNA-Sequencing Analysis Revealed Expression of a Novel Stay-Green Gene from Zoysia japonica (ZjSGR) Caused Chlorophyll Degradation and Accelerated Senescence in Arabidopsis
Source: Front Plant Sci. 2016 Dec 16;7:1894. doi: 10.3389/fpls.2016.01894 (PMC5159421; doi:10.3389/fpls.2016.01894)
Supplement: Table S1 — Primers used for the RNA-seq data verification. [file Table1.DOC]

**Supplemental Table 1** Primers used for the RNA-seq data verification

| Primer name | Primer sequence (5'-3') |
| --- | --- |
| AtSEN1-F | GTCATCGGCTATTTCTCCACCT |
| AtSEN1-R | GTTGTCGTTGCTTTCCTCCATC |
| AtSAG21-F | GGAAGAATCAACCCAGAAGA |
| AtSAG21-R | GCACCAGATCATTACCCACT |
| AtSAG14-F | ATGTGGCAGTTGTATCAGAAGC |
| AtSAG14-R | GGTGTTTAGCATAATTTTGACCGGA |
| AtNYC1-F | CAAGTCTTGGAGCACCGTCTC |
| AtNYC1-R | CCACGACCCATTTCCCTTAA |
| AtYUC9-F | GACGGAGTTTGACGGAGAAG |
| AtYUC9-R | CCCTCGGTAAAACATGAACC |
| AtIAA6-F | GAAGAGTCAAGCGGTGGGATG |
| AtIAA6-R | TTGCTCGAACCAAGGTCAAT |
| AtIAA29-F | CGAGTCCCTCACTAACTCCTTG |
| AtIAA29-R | AGTAGCCAGTCACCCTCTTTCC |
| AtNAC29-F | TACAGACAAAGCCATTCACAGC |
| AtNAC29-R | AACCGTTACGTTTCGTTGATGC |
| AtWRKY6-F | GTTGCTATCCGGGTCAATGTCT |
| AtWRKY6-R | GGAGGTGAGTGGGTGAGGTCTA |
| AtNAC47-F | AAGCAACCGGAACAGATAAATT |
| AtNAC47-R | ACCACCGCTCCTAGAAGAGTTA |
