# Supplementary material for: Functional and RNA-Sequencing Analysis Revealed Expression of a Novel Stay-Green Gene from Zoysia japonica (ZjSGR) Caused Chlorophyll Degradation and Accelerated Senescence in Arabidopsis
Source: Front Plant Sci. 2016 Dec 16;7:1894. doi: 10.3389/fpls.2016.01894 (PMC5159421; doi:10.3389/fpls.2016.01894)
Supplement: Table S2 — Cis-regulatory elements of the 5′ up-stream sequence of ZjSGR. [file Table2.DOCX]

**Supplemental Table 2 *Cis*-regulatory elements of the 5’ up-stream sequence of *ZjSGR***

| Site name | Organism | Position | Strand | Matrix score | Sequence | Function |
| --- | --- | --- | --- | --- | --- | --- |
| ARE | Zea mays | 260 | + | 6 | TGGTTT | cis-acting regulatory element essential for the anaerobic induction |
| CGTCA-motif | Hordeum vulgare | 496 | - | 5 | CGTCA | cis-acting regulatory element involved in the MeJA-responsiveness |
| AT1-motif | Solanum tuberosum | 420 | - | 13 | AATTATTTTTTATT | part of a light responsive module |
| Box 4 | Petroselinum crispum | 289 | + | 6 | ATTAAT | part of a conserved DNA module involved in light responsiveness |
| Box I | Pisum sativum | 145 | + | 7 | TTTCAAA | light responsive element |
| CAAT-box |  |  |  |  |  | common cis-acting element in promoter and enhancer regions |
| CATT-motif | Zea mays | 64 | - | 6 | GCATTC | part of a light responsive element |
| EIRE | Nicotiana tabacum | 156 | - | 7 | TTCGACC | elicitor-responsive element |
| F-box | Lycopersicon esculentum | 334 | - | 10 | CTATTCTCATT |  |
| GATA-motif | Arabidopsis thaliana | 129 | + | 7 | GATAGGA | part of a light responsive element |
| GC-motif | Zea mays | 519 | + | 6 | CCCCCG | enhancer-like element involved in anoxic specific inducibility |
| HD-Zip 1 | Arabidopsis thaliana | 221 | + | 8.5 | CAAT(A/T)ATTG | element involved in differentiation of the palisade mesophyll cells |
| HD-Zip 2 | Arabidopsis thaliana | 221 | + | 8 | CAAT(G/C)ATTG | element involved in the control of leaf morphology development |
| HSE | Brassica oleracea | 440 | + | 9 | AAAAAATTTC | cis-acting element involved in heat stress responsiveness |
| MBS | Arabidopsis thaliana | 271 | + | 6 | TAACTG | MYB binding site involved in drought-inducibility |
|  | Arabidopsis thaliana | 676 | + | 6 | CAACTG | MYB binding site involved in drought-inducibility |
| MNF1 | Zea mays | 707 | + | 6.5 | GTGCCC(A/T)(A/T) | light responsive element |
| Sp1 | Oryza sativa | 522 | - | 6 | GGGCGG | light responsive element |
|  | Zea mays | 624 | + | 5.5 | CC(G/A)CCC | light responsive element |
| TATA-box |  |  |  |  |  | core promoter element around -30 of transcription start |
| TC-rich repeats | Nicotiana tabacum | 119 | - | 9 | GTTTTCTTAC | cis-acting element involved in defense and stress responsiveness |
|  | Nicotiana tabacum | 191 | + | 9 | ATTCTCTAAC | cis-acting element involved in defense and stress responsiveness |
| TCA-element | Nicotiana tabacum | 124 | - | 9 | CCATCTTTTT | cis-acting element involved in salicylic acid responsiveness |
| TCT-motif | Arabidopsis thaliana | 119 | - | 6 | TCTTAC | part of a light responsive element |
| TGA-element | Brassica oleracea | 543 | + | 6 | AACGAC | auxin-responsive element |
| TGACG-motif | Hordeum vulgare | 496 | + | 5 | TGACG | cis-acting regulatory element involved in the MeJA-responsiveness |
| AAGAA-motif | Avena sativa | 27 | + | 9 | gGTAAAGAAA |  |
|  | Avena sativa | 234 | + | 9 | gGTAAAGAAA |  |
